# Abortion without Suspicion of Pregnancy

**Published:** 1853-02

**Authors:** 


### SELECTIONS

FROM

### FOREIGN AND HOME MEDICAL JOURNALS.

---

*Abortion without a suspicion on the part of the Mother of the existence of Pregnancy.*—Case reported by Dr. Storer.—*August* 28. At 1 o'clock, this morning, Mrs. S., of Charlestown, was awakened by hemorrhage from the vagina. She was married the latter part of December last, but her catamenia have appeared regularly; she has never experiened the slightest nausea; her appetite has not been impaired; there has been no derangement whatever of the alimentary canal, nor perceptible enlargement of the abdomen.

Not having the slightest idea of her being pregnant, she was not a little alarmed, and aroused her husband, who at once called up her mother, with whom she resided. The mother had never thought her daughter pregnant, although constantly with her, and, at first, could not imagine the cause of the hemhorrhage. But when pains supervened, and assumed a degree of regularity in their recurrence, she felt satisfied that her daughter was in labor, and Dr. S. was sent for. In an hour or two after the commencement of the pains, the fœtus was expelled. It measured nine inches in length, and the umbilicus was half an inch from the centre of the body. Dr. S. supposed the mother to have been between four and five months pregnant—nearly five. Upon examining patient's breasts, they were found to be large, but she did not perceive any unusual fulness. The areolæ, however, exhited those changes always so reliable; their color was much deeper; their feel, velvety; and the glandular follicles much enlarged;—thus pointing out, not merely the pregnancy but also its advancement.

In a medico-legal point of view, this case is interesting; inasmuch as, from the rational signs of pregnancy, a fœtus may be carried even to the time most women quicken, without the fact being suspected by the bearer, or any of her friends.

